# The Correlation between Matrix Metalloproteinase-9 Point-of-Care Immunoassay, Tear Film Osmolarity, and Ocular Surface Parameters

**DOI:** 10.1155/2022/6132016

**Published:** 2022-04-11

**Authors:** Min-Ji Kang, Hyun Seung Kim, Man Soo Kim, Eun Chul Kim

**Affiliations:** ^1^Department of Ophthalmology, Sanggye Paik Hospital, College of Medicine, Inje University, Seoul, Republic of Korea; ^2^Department of Ophthalmology, Seoul St. Mary's Hospital, College of Medicine, The Catholic University of Korea, Seoul, Republic of Korea; ^3^Department of Ophthalmology, Eunpyeong St. Mary;s Hospital, College of Medicine, The Catholic University of Korea, Seoul, Republic of Korea; ^4^Department of Ophthalmology, Bucheon St. Mary's Hospital, The Catholic University of Korea, Bucheon, Republic of Korea

## Abstract

**Background:**

Dry eye disease is a multifactorial disease that is difficult to diagnose due to multiple causative factors. The study aimed to evaluate the correlations between tear film matrix metalloproteinase-9 (MMP-9), tear film osmolarity, and ocular surface parameters in patients with dry eyes.

**Methods:**

We performed a retrospective chart review for patients diagnosed with dry eye and investigated if associations existed amongst noninvasive tear breakup time (NIBUT); corneal staining scores; and MMP-9 grade, tear film osmolarity, and Schirmer's test I results.

**Results:**

Twenty-four eyes of 24 patients were enrolled in the current study. The grade of MMP-9 (0–4) was positively correlated with tear film osmolarity (*p*=0.027). However, neither qualitative (positive or negative) nor quantitative (grade 0–4) measurements of MMP-9 correlated with any other ocular surface parameters. The osmolarity in the positive corneal staining group was significantly higher than that in the negative group (321.6 ± 19.261 and 299.89 ± 16.213, respectively; *p*=0.018). None of the other ocular surface parameters were correlated with tear film osmolarity.

**Conclusion:**

Tear film MMP-9 may be an indicator for tear film osmolarity, or vice-versa. Moreover, osmolarity may have a correlation with corneal staining in patients with dry eye. Tear film MMP-9 and osmolarity tests can be helpful and convenient evaluation tools for identifying inflammation in dry eye disease in clinical practice.

## 1. Introduction

Dry eye disease is a multifactorial disease that is difficult to diagnose due to multiple causative factors [1, 2]. Schirmer's test I, tear breakup time, corneal and conjunctival staining scores, meibomian gland scoring, and symptom questionnaires are commonly used to evaluate dry eye disease [3, 4]. Additionally, two commercially available objective dry eye tests, including tear film matrix metalloproteinase-9 (MMP-9) and tear film osmolarity, can also be used to diagnose dry eye. Inflammation is increasingly recognised as a fundamental element in the pathogenesis of dry eye disease. MMP-9 and osmolarity are known players in the inflammatory pathways that are involved in dry eye disease [5–7]. Therefore, commercially available MMP-9 and osmolarity tests have been introduced to facilitate the diagnosis of dry eye in the clinic.

MMP-9 is traditionally measured in laboratories by enzyme-linked immunosorbent assay (ELISA) or proteomic technologies, but it is not widely evaluated in clinical settings because of its inconvenience; that is, it is time consuming and requires a non-point-of-care setting [8, 9]. However, the InflammaDry® is a noninvasive, single-use, and commercially available point-of-care assay that was developed to conveniently measure both active and latent MMP-9 in the clinic [1, 4, 9]. InflammaDry represents positive results when MMP-9 levels exceed 40 ng/mL [1, 9, 10].

In the past, the TearLab Osmolarity System was the only commercially available device that could measure tear film osmolarity. The TearLab device uses microelectrodes and noninvasively measures the electrical impedance of a 50 nL tear sample [2, 11, 12]. However, the novel I-PEN® Osmolarity System was recently introduced for commercial use. I-PEN® also uses electrical impedance for osmolarity measurements, but unlike the TearLab system, the I-PEN® Osmolarity System does not require the transfer of tear samples to a separate measurement unit and it can be operated with smaller handheld devices [11].

The relationship between MMP-9 and osmolarity has not been widely evaluated, and discrepancies exist in the literature. Li et al. [13] and VanDerMeid et al. [6] reported that MMP-9 was correlated with osmolarity. However, Schargus et al. [4] reported that MMP-9 and osmolarity had no correlation. Therefore, the present study aimed to evaluate the correlations between InflammaDry MMP-9, handheld I-PEN tear film osmolarity, and other ocular surface parameters, including Schirmer's test I, noninvasive tear break up time (NIBUT), and corneal staining scores in patients with dry eye.

## 2. Materials and Methods

### 2.1. Study Design

In this study, we retrospectively reviewed the medical charts of patients with dry eyes from June 1, 2018 to June 30, 2018. This study was approved by the Institutional Review Board at the Bucheon St. Mary's Hospital and followed the tenets of the Declaration of Helsinki.

### 2.2. Participants

Patients who were diagnosed with dry eye were included in this study, and dry eye was defined as an Ocular Surface Disease Index (OSDI) score ≥13, and one or more of the following values: (1) NIBUT, <10 s; (2) osmolarity, >308 mOsm/L in either eye or interocular difference >8 mOsm/L; or (3) a positive corneal staining score according to Tear Film and Ocular Surface Society (TFOS) Dry Eye Workshop (DEWS) II definition [14]. Patients older than 18 years old were enrolled in the study, and only the right eye was included for evaluation. Patients were excluded if they had allergic conjunctivitis, uveitis, pseudoexfoliation syndrome, keratoconus, or herpetic keratitis; wore contact lenses within one month of the study; underwent ocular surgery within three months of the study; used corticosteroids, immunosuppressants, antiglaucoma medications, cyclosporine, or antihistamines; or were diagnosed with autoimmune diseases, including Sjogren syndrome, Stevens–Johnson syndrome, and graft-versus-host diseases.

### 2.3. Clinical Assessments

We examined NIBUT; corneal staining scores; and tear film osmolarity, MMP-9 levels, and Schirmer's test I results.

Tear film osmolarity was examined using the I-PEN® Osmolarity System (I-MED Pharma Inc., Dollard-des-Ormeaux, Quebec, Canada). First, the I-PEN single-use sensor was inserted into the device, and the patients were asked to close their eyes for 30–60 s. Next, the patients were instructed to open their eyes, and the sensor tip was placed onto the palpebral conjunctiva, and the operator ensured that both gold nodes of the sensor were in good contact with the palpebral conjunctiva. After positioning, the I-PEN made an audible beep and displayed the osmolarity on its screen [11].

The MMP-9 test was performed with the InflammaDry test kit (Rapid Pathogen Screening Inc., Sarasota, FL, USA). Briefly, a sample fleece was gently dabbed onto the lower palpebral conjunctiva at multiple locations several times to saturate the fleece with tears. Next, the saturated sample fleece was gently placed onto the transfer window of a cassette body and pressed firmly until it was properly assembled. The absorbent tip of the assembled test unit was placed into a buffer solution for 20 s, and the test unit was placed on a flat surface for 10 min. After 10 min, we evaluated the window for MMP-9 results. A blue line represented a valid test; therefore, if the blue line was not presented, the test was deemed invalid. A red line represented positive results and indicated a concentration of MMP-9 that was ≥40 ng/mL. However, the presence of a blue line and the absence of a red line represented negative results and indicated that the concentration of MMP-9 was <40 ng/mL [1, 4, 9]. The positive red lines were graded subjectively according to the grading index, which is classified with different intensities by an experienced investigator (ECK) as follows: trace positive, weak-positive, positive, and strong positive [1].

Schirmer's test I was performed without anaesthesia. Briefly, a sterile strip (Eagle Vision, Memphis, TN, USA) was inserted into the lateral third of the lower eyelid for 5 min while the patients' eyes were closed. After 5 min, the wetted length was measured.

The Oculus Keratograph 5 M (Oculus, Wetzlar, Germany) was used to measure the NIBUT. Localised breaks in the tear film were detected by infrared waves in real-time without the use of fluorescein strips. Patients were instructed to blink twice to stabilise the film. Thereafter, the patients were asked to refrain from blinking and stare into the central light. During the measurement, 22 rings with >1,000 measurement points per ring were projected onto the cornea. This resulted in 22,000 analysed data points per frame. The breakup points appeared on the grid map, and the video recording was stopped after 25 s or until the next blink. The time from the first appearance of a break of tear film was recorded as the NIBUT [15, 16].

Corneal staining scores were assessed according to the Oxford grading scale [17]. After staining with fluorescein punctate corneal epithelial, erosions were scored from 0 (absent) to 5 (severe) according to a reference figure [17] by a single investigator (ECK).

### 2.4. Statistical Analyses

Statistical analyses were performed using SPSS software (version 22.0, IBM Corp, Armonk, NY, USA). First, normality analyses were performed using the Kolmogorov–Smirnov test, and comparisons between two groups were performed with independent *t*-tests for normally distributed data and Mann–Whitney tests for non-normally distributed data. Next, correlation analyses were performed using Pearson correlation tests for normally distributed and Spearman rank tests for non-normally distributed data. The results are presented as the mean ± standard deviation (SD). For all analyses, *p* < 0.05 was considered statistically significant.

## 3. Results

Overall, 24 eyes of 24 patients were enrolled in this study. The baseline characteristics of the patients are described in Table 1. The mean age of participants (17 female (70.8%)) was 48.33 ± 14.80 years.

### 3.1. MMP-9 and Osmolarity

Of the 24 eyes, 19 eyes (79%) were MMP-9 positive and five eyes (21%) were MMP-9 negative (Table 2). Tear film osmolarity was higher in the MMP-9 positive group than in the MMP-9 negative group (315.95 ± 22.205 and 304.0 ± 11.269, respectively); however, this difference was not statistically significant (*p*=0.302) (Table 2).

**Figure 1:**
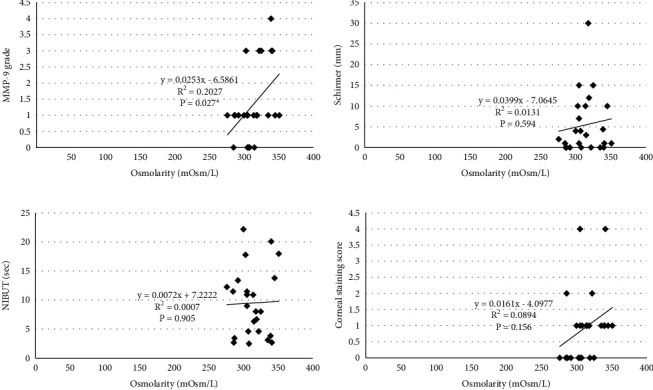
Correlation between osmolarity and ocular surface parameters. (a) MMP-9 grade, (b) Schirmer's test I, (c) NIBUT, and (d) corneal staining scores. MMP-9 = matrix metalloproteinase-9, NIBUT = noninvasive tear break up time, and *R* = correlation coefficient. ^*∗*^Indicates statistical significance (*p* < 0.05).

However, we observed a statistically significant positive correlation between MMP-9 grade and osmolarity (*p*=0.027) after we subdivided patients into MMP-9 grades 0–4 (Table 3 and Figure 1(a)).

### 3.2. MMP-9 and Ocular Surface Parameters

When we evaluated qualitative (positive or negative) results of MMP-9 and other ocular surface parameters including Schirmer, NIBUT, and corneal staining score, there was no statistical significance between MMP-9 positive and negative groups (Table 2). Additionally, the grade of MMP-9 (0–4) was not correlated with any other parameters (Table 3).

Next, we divided each of the ocular surface parameters into two groups according to dry eye severity: the positive and negative corneal staining score groups; the <10 and ≥10 mm Schirmer test I groups; and the <5 and ≥5 s NIBUT groups (Table 4) and compared differences in MMP-9 grade between each group. There were no significant differences in MMP-9 grade between the positive and negative corneal staining score groups, <10 and ≥10 mm Schirmer test groups, or <5 and ≥5 s NIBUT groups (Table 4).

SD = standard deviation; MMP-9 = matrix metalloproteinase-9.

### 3.3. Osmolarity and Ocular Surface Parameters

**Figure 2:**
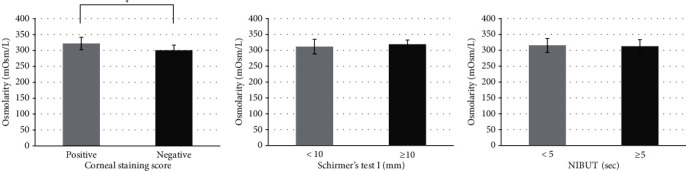
Comparison of osmolarity between ocular parameter groups stratified by dry eye severity. (a) Corneal staining scores, (b) Schirmer's test I, and (c) noninvasive tear break up time (NIBUT). ^*∗*^Indicates statistical significance (*p* < 0.05).

There were no correlations between osmolarity and any other ocular surface parameters, including Schirmer's test I results, NIBUT, and corneal staining scores (Table 5 and Figure 1). However, when we compared osmolarity with ocular surface parameter groups according to dry eye severity qualitatively (e. g., positive/negative corneal staining score, <10/≥10 Schirmer test I, and <5/≥5 NIBUT), the osmolarity value in the positive corneal staining group was significantly higher than that in the negative corneal staining group (321.6 ± 19.261 and 299.89 ± 16.213, respectively; *p*=0.018) (Table 4 and Figure 2). However, there were no differences in osmolarity between the <10 and ≥10 mm Schirmer test I groups or the <5 and ≥5 s NIBUT groups.

## 4. Discussion

Dry eye is usually difficult to diagnose and sometimes undiagnosed because of multiple causative factors and a weak correlation between symptoms and common clinical and evaluation assessments [18–20]. However, studies have confirmed that inflammation is an important factor that is associated with dry eye, and there have been many attempts to diagnose dry eye using inflammatory markers and parameters [5, 21, 22].

MMP-9 is an inflammatory marker that is increased in the tears of patients with dry eyes [9, 23]. Inflammation activates the production of MMP-9, which triggers inflammatory pathways, such as the stress-activated protein kinase (SAPK) signalling cascade, ultimately escalating inflammation [24, 25]. MMP-9 plays a role in corneal extracellular matrix remodelling during inflammation [26, 27]; however, it can also promote inflammation by cleavage of precursors of proinflammation factors [28, 29]; therefore, MMP-9 can promote corneal extracellular matrix degradation and epithelial cell loss [9, 30].

In 1992, tear osmolarity was considered the gold standard diagnostic test for dry eye by Farris [31], and multiple studies that focused on tear film osmolarity in dry eyes have been performed. Studies have reported that hyperosmolarity activates inflammation pathways and promotes the release of proinflammatory cytokines, which subsequently provokes corneal epithelial damage and goblet cell loss [32, 33]. Additionally, inflammation can reduce tear film stability and further increase osmolarity [34, 35].

In our study, MMP-9 grade was positively correlated with tear film osmolarity. Previous studies evaluated the correlation between tear film osmolarity and MMP-9 using InflammaDry and ELISA, both of which reported that MMP-9 and tear film osmolarity were not significantly correlated [4]. However, they also mentioned that positive MMP-9 test results were associated with elevated tear film osmolarity. Therefore, there may be a link between MMP-9 and osmolarity in patients with more advanced dry eye disease [4]. Conversely, results from other studies suggest that MMP-9 is correlated with osmolarity. For example, Li et al. [13] investigated corneal epithelial cells and reported that the expression of MMP-9 increased as the osmolarity of the media increased. Similar to our results, VanDerMeid et al. [6] reported that MMP-9 extracted from Schirmer strips correlated well with tear osmolarity. Hyperosmolarity in tears can trigger the SAPK pathway and lead to MMP-9 release from corneal epithelial cells, and this can initiate a cycle of progressive inflammation [10, 25].

In the current study, MMP-9 was not correlated with any other ocular surface parameters, including Schirmer's test I, NIBUT, and corneal staining scores, and previous studies that have evaluated the correlation between MMP-9 and ocular surface parameters have had varying results. For example, some studies reported that there was a statistically significant correlation between MMP-9 and these ocular surface parameters [1, 9]; however, other studies revealed that none of these parameters were correlated with MMP-9 [4, 6, 36, 37]. Overall, the correlation between MMP-9 and dry eye signs is not yet definitively established; therefore, further research should be conducted to verify this relationship.

Additionally, results from previous studies that evaluated the correlation between tear film osmolarity and corneal staining scores have reported varying results. For example, Mathews et al. [7] and Kook et al. [37] reported that higher tear osmolarity is associated with higher corneal staining scores, whereas other studies reported that tear osmolarity is not correlated with corneal staining scores [4, 38]. In our study, tear film osmolarity was not correlated with corneal staining scores; however, the osmolarity in the positive corneal staining group was significantly higher than that in the negative group. Thus, although the degree of osmolarity was not correlated with the degree of corneal staining, the positive result of corneal staining itself may be associated with hyperosmolarity.

Further, the Schirmer test results and NIBUT were not correlated with tear film osmolarity in this study. Similarly, previous studies reported that Schirmer test results and tear breakup time were not significantly correlated with tear film osmolarity [4, 6, 7]. However, recently, Park et al. [39] reported that tear osmolarity measured by I-PEN was negatively correlated with tear breakup time and the Schirmer test, and Kook et al. [37] reported that tear osmolarity was negatively correlated with BUT and the Schirmer test. Further studies are needed to resolve the discordance among these reports.

In the past, tear film osmolarity and MMP-9 were difficult to measure in clinical settings. However, InflammaDry and TearLab or I-PEN can be used to measure MMP-9 and tear film osmolarity, respectively, in clinical settings. Commonly evaluated clinical signs and symptoms, such as Schirmer's test, tear breakup time, corneal and conjunctival staining scores, meibomian gland evaluations, and the ocular surface disease index (OSDI), do not always correlate well with dry eye, making the diagnosis of dry eye difficult [18–20]. Therefore, supplemental measurements that evaluate inflammation, such as MMP-9 and tear film osmolarity, can help to diagnose dry eye in clinical practice.

This study is limited by its retrospective study design and a small sample size. Furthermore, the subjective nature of the quantitative evaluation of MMP-9 grading has its limitations. Therefore, further prospective studies with larger sample sizes and more objective MMP-9 grading evaluations should be conducted to verify our results.

## 5. Conclusions

MMP-9 may be an indicator for tear film osmolarity and vice-versa. In addition, osmolarity may have a correlation with corneal staining in patients with dry eyes. Overall, our findings suggest that MMP-9 and tear film osmolarity evaluations may be used to easily and conveniently identify inflammation in patients with dry eye.

## Figures and Tables

**Table 1:** Baseline characteristic of patients enrolled in the study.

	Mean ± SD
Patients, n	24
Age, years	48.33 ± 14.80 (range, 25 to 76)
Sex (n, %)	Male (7, 29.2) and female (17, 70.8)
Corneal staining score	0.96 ± 1.09 (range, 0 to 4)
Schirmer's test I (mm)	5.43 ± 7.07 (range, 0 to 30)
Noninvasive tear break up time (NIBUT) (sec)	9.48 ± 5.73 (range, 2.48 to 22.18)
Osmolarity (mOsmol/L)	313.45 ± 20.36 (range, 276 to 351)
InflammaDry (MMP-9) test (positive/negative)	19/5

SD = standard deviation; MMP-9 = matrix metalloproteinase-9.

**Table 2:** Qualitative evaluation of MMP-9 with ocular surface parameters.

	MMP-9 positive	MMP-9 negative	*p* value
Eyes, n	19	5	
Osmolarity (mOsmol/L)	315.95 ± 22.205	304.0 ± 11.269	0.302
Schirmer's test I (mm)	5.65 ± 7.650	4.6 ± 6.025	0.942
NIBUT (sec)	10.07 ± 6.195	7.26 ± 4.0721	0.393
Corneal staining score	0.89 ± 0.994	1.2 ± 1.643	0.939

MMP-9 = matrix metalloproteinase-9; NIBUT = noninvasive tear break up time. ^*∗*^Indicates statistical significance (*p* < 0.05)

**Table 3:** Correlation of MMP-9 grade with ocular surface parameters.

	MMP-9 grade	
	R correlation coefficient	*p* value
Osmolarity (mOsmol/L)	0.45	0.027^*∗*^
Schirmer's test I (mm)	−0.007	0.976
NIBUT (sec)	0.036	0.868
Corneal staining score	0.111	0.607

MMP-9 = matrix metalloproteinase-9; NIBUT = noninvasive tear break up time. ^*∗*^Indicates statistical significance (*p* < 0.05).

**Table 4:** Comparison of MMP-9 grade and osmolarity between ocular surface parameter groups according to dry eye severity.

	MMP-9 grade		Osmolarity (mOsmol/L)	
	Mean ± SD	*p* value	Mean ± SD	*p* value
Corneal staining score				
Positive	1.4 ± 1.242	0.768	321.6 ± 19.261	0.018^*∗*^
Negative	1.22 ± 1.093		299.89 ± 16.213	
Schirmer's test I (mm)				
<10	1.29 ± 1.213	0.70	311.41 ± 23.071	0.391
≥10	1.43 ± 1.134		318.43 ± 14.07	
NIBUT (sec)				
<5	1.63 ± 1.506	0.589	315.63 ± 22.155	0.624
≥5	1.19 ± 0.981		312.38 ± 20.746	

SD = standard deviation; MMP-9 = matrix metalloproteinase-9; NIBUT = noninvasive tear break up time. ^*∗*^Indicates statistical significance (*p* < 0.05).

**Table 5:** Correlation between osmolarity and ocular surface parameters.

	Osmolarity (mOsmol/L)	
	R correlation coefficient	*p* value
MMP-9 grade	0.45	0.027^*∗*^
Schirmer's test I (mm)	0.115	0.594
NIBUT (sec)	0.026	0.905
Corneal staining score	0.299	0.156

MMP-9 = matrix metalloproteinase-9; NIBUT = noninvasive tear break up time. ^*∗*^Indicates statistical significance (*p* < 0.05).

## Data Availability

The datasets used and analysed during the current study are available from the corresponding author upon reasonable request.
